# Effect of DODAB Nano-Sized Cationic Bilayer Fragments against *Leishmania amazonensis*

**DOI:** 10.3390/molecules25235741

**Published:** 2020-12-05

**Authors:** Thalita C. S. Ferreira, Ismael P. Sauter, Lina Borda-Samper, Enyd Bentivoglio, Jarina P. DaMata, Noemi N. Taniwaki, Patrício R. Orrego, Jorge E. Araya, Nilton Lincopan, Mauro Cortez

**Affiliations:** 1 Departamento de Parasitologia, Instituto de Ciências Biomédicas, Universidade de São Paulo, São Paulo, SP 05508-000, Brazil; ferreira.tcs@outlook.com (T.C.S.F.); ipsauter@gmail.com (I.P.S.); linaborda@gmail.com (L.B.-S.); damatajp@yahoo.com (J.P.D.); 2 Departamento de Microbiologia, Instituto de Ciências Biomédicas, Universidade de São Paulo, São Paulo, SP 05508-000, Brazil; enyd.bentivoglio@gmail.com; 3 Instituto Adolfo Lutz, São Paulo, SP 02146-000, Brazil; ntaniwak@hotmail.com; 4 Facultad de Ciencias de la Salud, Departamento Biomédico, Universidad de Antofagasta, Antofagasta, CP 1270300, Chile; patricio.orrego@uantof.cl; 5 Facultad de Ciências de la Salud, Departamento de Tecnología Médica, Universidad de Antofagasta, Antofagasta, CP 1270300, Chile; jorge.araya@uantof.cl

**Keywords:** antileishmanial activity, cationic bilayer fragments, dioctadecyldimethylammonium bromide (DODAB), *Leishmania amazonensis*

## Abstract

The dioctadecyldimethylammonium bromide (DODAB) is a double-chained cationic lipid with potent bactericide and fungistatic activities; however, its toxicity on protozoan parasites is still unknown. Here, we show the antileishmanial activity of DODAB nano-sized cationic bilayer fragments on stationary-phase promastigotes and amastigotes of *Leishmania amazonensis*, the causative agent of cutaneous leishmaniasis. Upon treatment with DODAB, we analyzed the parasite surface zeta-potential, parasite viability, cellular structural modifications, and intracellular proliferation. The DODAB cytotoxic effect was dose-dependent, with a median effective concentration (EC_50_) of 25 µM for both life-cycle stages, comparable to the reported data for bacteria and fungi. The treatment with DODAB changed the membrane zeta-potential from negative to positive, compromised the parasite’s morphology, affected the cell size regulation, caused a loss of intracellular organelles, and probably dysregulated the plasma membrane permeability without membrane disruption. Moreover, the parasites that survived after treatment induced small parasitophorous vacuoles and failed to proliferate inside macrophages. In conclusion, DODAB displayed antileishmanial activity, and it remains to be elucidated how DODAB acts on the protozoan membrane. Understanding this mechanism can provide insights into the development of new parasite-control strategies.

## 1. Introduction

The protozoan parasites *Leishmania* spp. are the etiologic agents of the leishmaniasis, a group of poverty-associated diseases endemic in more than 98 countries [1], with one million new cases reported annually [2]. During their life-cycle, they undergo drastic morphological changes, presenting two main stages: the amastigotes (non-motile forms that multiply mainly inside the phagocytic immune cells of mammalian hosts) and promastigotes (flagellated extracellular stage found in the gut of the sand fly vector) [1]. The clinical manifestation is mostly dependent on host-parasite interactions and environmental factors, ranging from self-healing skin lesions to visceral disease [3,4]. Current chemotherapy, mainly with pentavalent antimonials, amphotericin B, and miltefosine, presents a series of limitations—for instance: high costs, low efficacy, poor safety, the emergence of parasite resistance, administration route, and length of treatment [5]. It is noteworthy that, despite the many efforts of the World of Health Organization in improving current treatments, only a few available candidates have shown some degree of therapeutic effect, including aminopyrazole, pyrazolopyrimidine, oxaborole, and nitroimidazole compounds [6,7]. Concurrently, there are few validated targets for drug discovery in *Leishmania* [8]. In this context, the discovery of more efficient therapeutics and new potential targets is a priority in leishmaniasis research.

Dioctadecyldimethylammonium bromide (termed DODAB) is a double-chained cationic lipid that self-assembles as bilayer vesicles in water medium and has membrane-mimicking properties [9]. Such biophysical properties allow DODAB liposomes to be used as a drug delivery system [10,11] and a DNA carrier system [12,13]. Furthermore, DODAB by itself presents immunoadjuvant [14,15] and antimicrobial effects [16,17]. The in vitro toxicity of DODAB has been reported for mammalian cells [18,19], as well as for several pathogenic strains of bacteria and fungi [16,17], but so far there are no reported data on its effect on protozoan parasites.

Since DODAB shows toxic effects against different pathogens, we enquired whether *Leishmania* parasites would be affected by cationic nano-sized fragments of this compound. Thus, the present study aims to investigate the effect of DODAB bilayer fragments on the stationary-phase promastigotes and axenic amastigotes of *L. amazonensis*.

## 2. Results

### 2.1. Electrophoretic Mobility of L. amazonensis Promastigotes after DODAB Treatment

**Figure 1:**
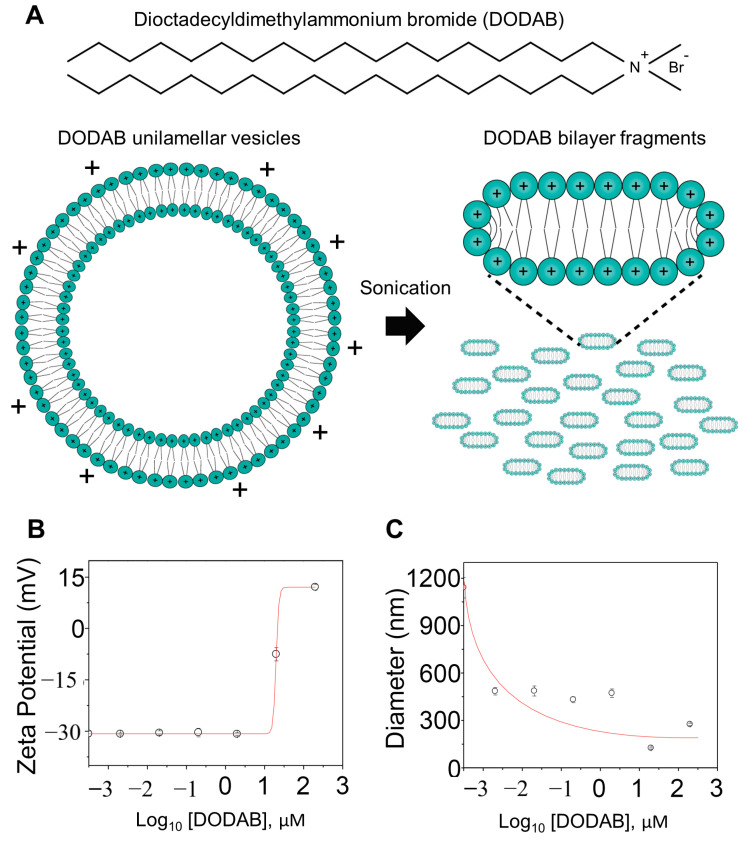
Particle size and zeta potential of *Leishmania* promastigotes after dioctadecyldimethylammonium bromide (DODAB) treatment. (**A**) Schematics of DODAB chemical structure (C_38_H_80_NBr) and DODAB bilayers assembly in aqueous dispersions. The interaction between DODAB (0, 0.002, 0.02, 0.2, 2, 20, 200 µM) and promastigote cells (1 × 10^6^ parasites/mL) was analyzed after 2 h of incubation at 26 °C in 0.264 M D-glucose solution (IGP buffer). (**B**) The charge of the parasites increased from −30.72 ± 0.79 to 14.15 ± 1.06 mV at 200 µM. (**C**) Non-treated promastigotes had a mean cell diameter of 1140 ± 65 nm, whereas the 200 µM DODAB pre-treated promastigotes had a mean cell diameter of 281.6 ± 6.5 nm.

DODAB tends to assemble into large unilamellar vesicles in water solutions, but nano-sized cationic bilayer fragments can be obtained by sonication [20], as shown in Figure 1A. The electrostatic repulsion at a low ionic strength keeps the bilayer fragments stable in aqueous dispersions [20]. The parasites were incubated for two hours with a DODAB bilayer fragment suspension in a 0.264 M D-glucose solution (IGP buffer), as previously reported [17]. Notably, the surface charge of promastigotes changed from negative (−30.72 ± 0.79 mV) to positive (14.15 ± 1.06 mV) after 200 µM of DODAB treatment (Figure 1B). Additionally, there was a reduction in the promastigote’s size from 1140 ± 65 to 281.6 ± 6.5 nm at the highest concentration (Figure 1C). These data show that the DODAB cationic bilayers probably adsorb onto *L. amazonensis* promastigotes, changing the parasite membrane surface potential. Therefore, parasites are susceptible to drastic ionic disbalance upon treatment.

### 2.2. Effect of DODAB in the Infective Forms of L. amazonensis

**Figure 2:**
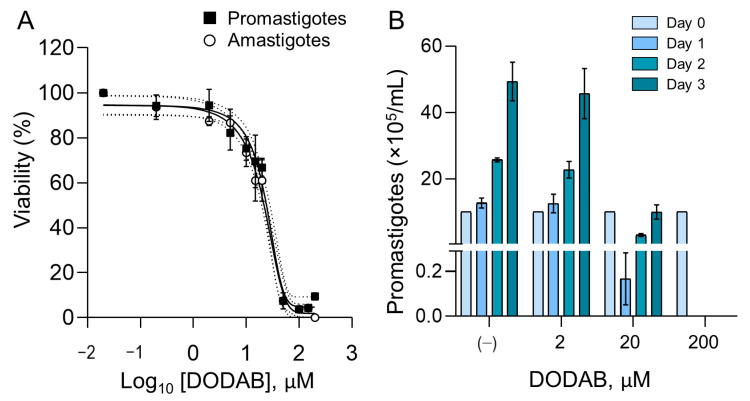
Viability of *Leishmania* after DODAB treatment. (**A**) The graph represents the percentage of viable *Leishmania* parasites (promastigote and amastigotes) measured by an MTT-based assay after treatment with different concentrations of DODAB (0.02, 0.2, 2, 5, 10, 15, 20, 50, 100, 150, and 200 µM) for 2 h. The data were normalized with the control parasites (incubation with IGP buffer only). Data points are means, and error bars represent the standard deviation from triplicates of two independent experiments. The solid line represents the predicted sigmoid dose–response curve and the dotted lines show the 95% interval confidence. (**B**) After the treatment of the parasites, the promastigotes were washed and incubated in complete parasite medium for recovery and multiplication at a density of 1 × 10^6^ parasites/mL (Day 0). The number of parasites was counted every day for 3 days. Bars are means, and the error bars represent standard deviation from triplicates. The “*y* axis” has two segments: 0–0.3 (bottom) and 0.3–60 (top).

We evaluated the parasite viability after treatment with different concentrations of DODAB bilayer fragment suspension. For both stationary-phase promastigotes and amastigotes, the concentrations of 2 and 20 µM reduced the parasite viability by 6% and 35%, respectively (Figure 2A). At 200 µM, the promastigotes’ viability decreased ~93%, with a total elimination of the amastigotes. The activity in terms of the 50% effective concentrations (EC_50_) was estimated at 25 µM for both forms. As promastigotes proliferate fast in culture, we investigated whether the parasites that survived after treatment could proliferate. Although most promastigotes were killed at a concentration of 20 µM, the remaining live parasites grew in complete medium. No parasite growth was observed at 200 µM, which was the highest tested concentration (Figure 2B).

### 2.3. Loss of Intracellular Structures in L. amazonensis Parasites Treated with DODAB

**Figure 3:**
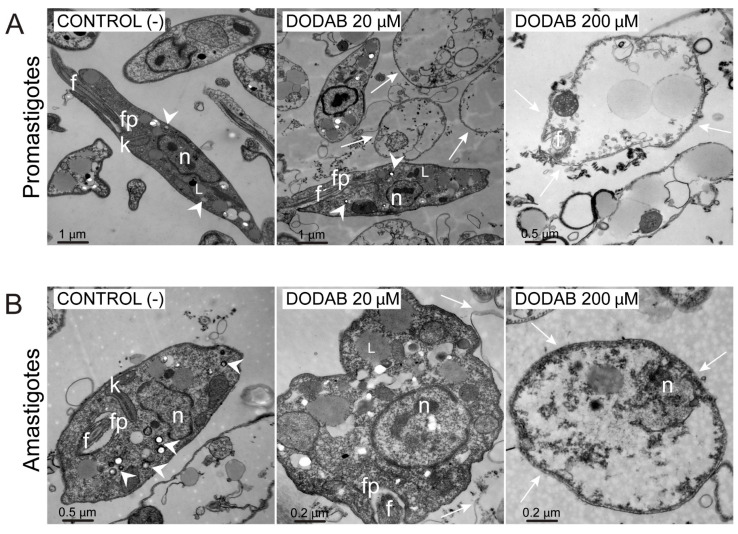
Ultrastructural analysis of *Leishmania* promastigotes (**A**) or amastigotes (**B**) treated with DODAB. Untreated parasites (control—IGP buffer) or those treated with 20 and 200 µM of DODAB for 2 h were washed and observed under transmission electron microscopy. DODAB altered the parasites’ cellular structures, with no disruption of the plasma membrane (white arrowheads). n: nuclei; fp: flagellar pocket; f: flagellum; k: kinetoplast; L: lipid droplets. Scale bar represents in microns (µm).

Transmission electron microscopy reveled striking changes in the cellular ultrastructure of DODAB-treated parasites (Figure 3). While the untreated parasites showed a typical morphology, with normal structures such as flagellar pockets (fp), kinetoplasts (k), and nuclei (n), (Figure 3A,B, image at the left), parasites treated with DODAB presented different degrees of cellular alterations. After treatment, the parasites became more rounded, demonstrating cell retraction. Additionally, the parasite membrane lost integrity without the rupture of its structure. At a concentration of 20 µM, some promastigotes displayed an increased number of lipid droplets, and others lost their overall intracellular organelles (Figure 3A, middle image). Amastigotes showed an abnormal swollen appearance, several vacuoles with different shapes, and the loss of acidocalcisome integrity (Figure 3B, middle image). The parasite intracellular integrity was abrogated at a 200 µM concentration. Together, the results indicate that DODAB causes significant morphological alterations to *L. amazonensis* promastigotes and amastigotes, from discrete changes to the complete loss of intracellular organelles.

### 2.4. DODAB Treatment Impairs L. amazonensis Proliferation in Macrophages

**Figure 4:**
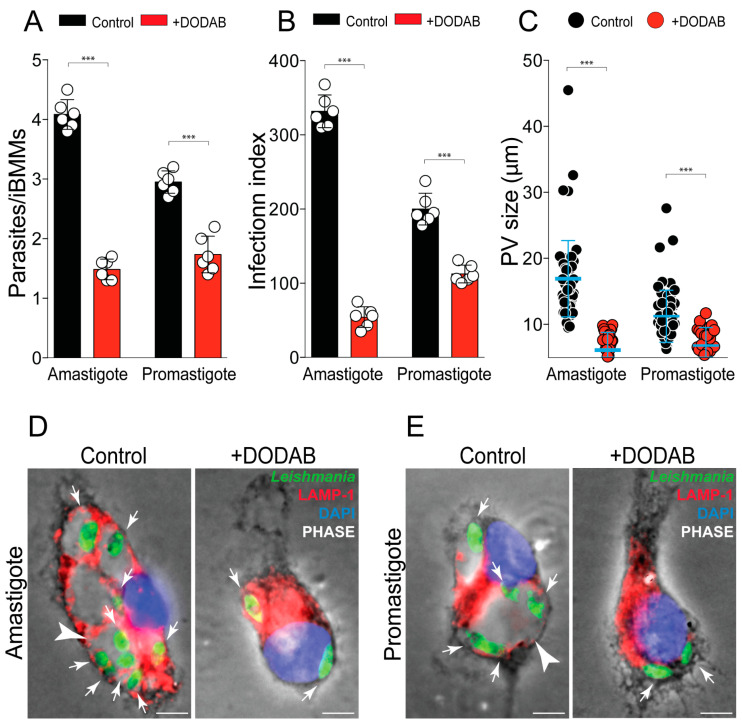
Pre-treatment with DODAB affects the *Leishmania* proliferation in macrophages. Untreated *L. amazonensis* amastigotes or promastigotes (control—IGP buffer) or pre-treated with 20 µM of DODAB for 2 h were used in infection assays with bone bone marrow-derived macrophages (BMM). Promastigotes (MOI 5) or amastigotes (MOI 2) were incubated with the cells for 2 h, followed by the removal of non-internalized parasites. After 2 and 48 h of infection, the samples were fixed and processed for immunofluorescence to quantify the number of parasites per cell. The data for 48 h of infection is shown. (**A**) Parasites/infected BMM (iBMMs) or (**B**) infection index were calculated as described in Materials and Methods. Bars are means, and error bars represent the standard deviations from triplicates (data points) of two independent experiments. Data points are means, and error bars represent the standard deviation from triplicates. **** *p*< 0.001 control versus DODAB treatment (Student’s *t*-test). (**C**) The size of the parasitophorous vacuoles (PV) was measured as described in Materials and Methods. Scatter plot with mean and standard deviation; each data point represents an individual PV (n = 33). **** *p* < 0.0001 Control versus DODAB 20 µM (one-way ANOVA). Representative immunofluorescence images showing *Leishmania* amastigotes (**D**), or promastigotes (**E**) inside of BMM (green; small arrows). PVs were stained with polyclonal antibody for the lysosome-associated membrane glycoprotein 1 (LAMP-1) detection (red), and cell nuclei were stained with 4′,6-diamidino-2-phenylindole (DAPI, in blue). Phase contrast is also shown. Arrows indicate *Leishmania* parasites and arrowheads, small PV.

To investigate whether DODAB also affects the capacity of *Leishmania* to survive and proliferate inside murine bone marrow-derived macrophages (BMM), we performed an in vitro infection with pre-treated stationary-phase promastigotes or amastigotes. We chose to treat parasites with 20 µM of DODAB, because ~75% of parasites survive at this concentration. After two hours of infection, the cells phagocytosed a similar number of DODAB pre-treated or untreated parasites, with approximately 2 parasites/infected BMMs. However, the DODAB treatment drastically reduced the parasite proliferation (Figure 4A,B). While the number of intracellular untreated parasites increased after 48 h, the ratio of two parasites/infected BMM persisted for the pre-treated cultures, indicating that the parasites are alive, but are unable to proliferate (Figure 4A,B). Interestingly, the immunofluorescence staining of the lysosomal-associated membrane protein-1 (LAMP-1) revealed the formation of small parasitophorous vacuoles (PV), uncharacteristic of *L. amazonensis* infection. The pre-treatment of amastigotes with DODAB reduced the diameter of *Leishmania*-PV, in comparison with the control untreated parasites, from 16.91 to 6.11 µm on average. A similar phenotype was observed in cells infected with DODAB pre-treated promastigotes, in which the *Leishmania*-PV decreased from 11.23 to 6.8 µm on average (Figure 4C). These effects are clearly visualized in the representative images (Figure 4D,E) that highlight pre-treated parasites inside smaller PV, relative to the control untreated parasites.

## 3. Discussion

The potent bactericide and fungistatic activities of DODAB have been previously demonstrated [17,21,22]. Here, we report that DODAB also presents activity against *L. amazonensis*. These findings represent the first report of DODAB activity against protozoan parasites.

A cytotoxic dose-dependent effect was observed against both life-cycle stages. When the EC_50_ for *L. amazonensis* parasites is compared to the reported data (see Table 1), it is clear that *L. amazonensis* is comparably susceptible to DODAB treatment to some bacterial and fungi strains. The in vitro cytotoxicity of DODAB is considerably higher, roughly 40 orders of magnitude, for pathogenic microorganisms (bacteria, fungi, and *Leishmania*) compared to mammalian cells. The absorption of DODAB cationic bilayers onto cells alters the membrane surface charge from negative to positive, inducing cell death, probably by hampering the function of essential transporters [22]. However, the biophysical explanation for the differential toxicity against pathogenic microorganisms and mammalian cells has never been investigated.

Regarding reference drugs, the EC_50_ of DODAB is comparable with the reported values for amphotericin B and miltefosine against *L. amazonensis* promastigotes, 1.6 and 14.4 µM, respectively [23]. However, such comparison should be interpreted cautiously, since there are substantial differences in the assay conditions. While the parasites were incubated for only 2 h with DODAB, the period of treatment for the reference compounds was 24 h. Moreover, low ionic strength conditions were used in the experiments to stabilize the DODAB bilayer fragment suspension, hence at physiological conditions the effect could be conflicting.

DODAB fragment characteristics have been explored to be used in different treatments [22,24,25], as well for the preparation of formulations containing amphotericin B or miconazole, which form large aggregated in solutions without the use of solvents [22,26]. The colloidal stability and optimization of cationic DODAB bilayer fragments (BF) have been achieved from systematic studies of turbidimetry and dynamic light scattering for sizing and zeta-potential analysis [19,25,27]. In this regard, at a low ionic strength charged bilayer fragments (BF) are colloidally stable due to electrostatic repulsion, whereas the DODAB-BF turbidity and mean hydrodynamic (zeta-average) diameter increase, maintaining the zeta-potential approximately constant. This stability, however, is low at a higher ionic strength of electrolyte solutions of mono (i.e., NaCl) and dihydrogen-phosphate (Na_2_HPO_4_) salts, where the colloid instability induced by salt could be associated with decreased electrostatic repulsion among fragments, contributing to the destabilization and fusion of DODAB-BF and an increase in bilayer packing due to the screening of the bilayer charges by salt. In this matter, the stability of DODAB fragments in topical preparations must be investigated for leishmaniasis treatment, especially in association with recognized leishmanicidal drugs, including the novel options under investigation [28].

Although *Leishmania* has two major different parasite forms, the promastigote in the sand fly and the amastigote in the mammalian host, the basic cellular architecture is conserved between the two *Leishmania* cell stages and among different *Leishmania* species [29,30]. In fact, the surface structure of *Leishmania* is composed mostly of a dense glycocalyx, which does not diverge in the total anionic molecules [31]. Therefore, since both *Leishmania* life-cycle stages keep a net negative surface charge [32,33], the leishmanicidal action of DODAB should be preserved against different species. In this regard, in a similar way as for other microorganisms, the interaction between DODAB and *L. amazonensis* most likely occurs by electrostatic attraction between the cationic bilayer fragments and anionic components (e.g., lipophosphoglycan and proteophosphoglycan) responsible for the negative surface charge of the parasite [32,34] As amastigotes recovered from lesions present a surface charge more negative than promastigotes or axenic amastigotes [35], the absorption of cationic bilayers fragments may be more effective in these highly virulent parasite forms.

Single-chained quaternary ammonium surfactants solubilize cytoplasmatic membranes, causing cell disruption [22,36]. DODAB, however, is a double-chained quaternary ammonium surfactant, and its bactericide and fungistatic mechanism of action is not related to cell lysis [22,36]. Accordingly, an ultrastructural analysis of *L. amazonensis* treated with DODAB revealed no disruption of the plasma membrane. Nevertheless, the treated parasites displayed a loss of intracellular organelles, including acidocalcisomes. The acidocalcisomes are electrodense compartments that play an important role in osmoregulation [37]. Therefore, the changes observed in promastigotes zeta-potential measurements could result from ion transport deregulation (H^+^, phosphate, Ca^2+^, among others) caused by the treatment.

*L. amazonensis* is well known for its large PV hosting several amastigotes [38]. The parasites that survived after DODAB treatment induced smaller parasitophorous vacuoles and were unable to proliferate inside macrophages. The survival and proliferation of *Leishmania* parasites rely on the modulation of macrophages’ immune response [39]. For instance, *L. amazonensis* amastigotes release extracellular microvesicles containing DNA fragments that induce the expression of the surface glycoprotein CD200 in the host cell [40,41]. This mechanism is essential for parasite replication and disease progression [40,41]. Whether the low proliferation rate of intracellular parasites pre-treated with DODAB is related with a deficient macrophage modulation mechanism, such as the low secretion of microvesicles, remains to be elucidated.

Our data are still preliminary, and further analysis is warranted to define how DODAB affects the membrane of the parasite. Given that some compounds depending on its properties display differential toxicity against different species [42], the effect of DODAB should be investigated on different species of *Leishmania.* Moreover, the type of *Leishmania* cell death should be determined by further ultrastructural analysis, the determination of mitochondrial membrane potential, and studies with biochemical markers [43].

In conclusion, DODAB can kill *Leishmania*, as demonstrated by the change in the membrane zeta-potential, the loss of intracellular organelles, the dysregulation of cell size, and the possible dysregulation of membrane permeability. Understanding the mechanism behind such effect can provide insightful information regarding the identification of new targets and the development of new anti-parasite strategies.

## 4. Materials and Methods

### 4.1. Parasite Culture

*Leishmania (Leishmania) amazonensis* (IFLA/BR/67/PH8) was isolated from lesions on C57BL/6 mice and then propagated as promastigotes at 26 °C in M199 medium (Vitrocell) supplemented with 40 mM of HEPES, 2.5 µg/mL of hemin, 10 mM of adenine, 2 mM of L-glutamine, 2 µg/mL of D-biotin, 100 U/mL of penicillin, 100 µg/mL of streptomycin, and 20% inactivated fetal bovine serum (FBS, from Vitrocell), at pH 7.2. Subcultures were prepared weekly at initial density of 5 × 10^5^ promastigotes/mL up to 6 passages. To generate axenic amastigotes, stationary-phase promastigote cultures were incubated at 2.5 × 10^7^/mL in M199 media containing 0.25% glucose, 0.5% trypticase, 40 mM sodium succinate (at pH 4.5), 20% FBS, and 5% penicillin/streptomycin at 32 °C for 7 days. Parasites were washed 3 times in phosphate-buffered saline (PBS) before use in experiments.

### 4.2. Obtainment of Cationic Bilayers Fragments of Dioctadecyldimethylammonium Bromide (DODAB)

To obtain cationic bilayer fragments of DODAB at 2.0 mM, DODAB lipids (99.9% pure, Sigma–Aldrich, St. Louis, USA) were dispersed in isotonic glucose phosphate buffer (IGP; 1 mM of potassium phosphate buffer, pH 7.0, supplemented with 287 mM of glucose as an osmoprotectant), using a titanium macrotip probe [21]. This procedure dispersed the amphiphilic powder in water using a high-energy input, which not only produced bilayer vesicles but also disrupted these vesicles, thereby generating open bilayer fragments [20]. The preparation was subsequently centrifuged (14,000 rpm at 4 °C, for 1 h) to remove any titanium particles. DODAB were analyzed at a concentration of 1 mM. Sizes (Dz), zeta-potentials (ζ), and polydispersity index were determined using the ZetaPlus-ZetaPotential Analyzer (Brookhaven Instruments Corporation, Holtsville, USA), equipped with a 677 nm laser and dynamic light scattering (PCS) at 90° for particle sizing.

### 4.3. Parasite Treatment

*L. amazonensis* parasites were treated with different concentrations of DODAB in IGP buffer for 2 h at 26 °C (promastigotes) or 34 °C (amastigotes). Control treatment was performed by incubating the parasite with IGP solution in the same conditions. Subsequently, parasites were washed 3 times with PBS. Additionally, after the promastigotes/DODAB interaction, the particle size and zeta-potential changes were monitored using a ZetaPlus-ZetaPotential Analyzer (Brookhaven Instruments Corporation, Holtsville, USA).

### 4.4. Viability Assay

The parasiticidal activity of DODAB was determined by incubating *L. amazonensis* promastigotes or amastigotes with different concentrations (0.2, 2, 20, 50, 100, 150, and 200 µM). The number of viable cells was determined by an MTT-based assay, as previously described [44]. Briefly, after 2 h of incubation with DODAB/IGP, 30 µL of 5 mg/mL MTT (3-[4,5-dimethyl-2-thiazolyl]-2,5- diphenyl-2H-tetrazolium bromide; Sigma-Aldrich) diluted in M199 medium was added and the cultures were kept at 26 °C (promastigotes) or 34 °C (amastigotes) for 4 h. The reaction was stopped by adding 30 µL of 20% sodium dodecyl sulfate to each well, and the absorbance at 550 nm was determined in a plate reader (POLARstar Omega, BMG Labtech, Ortenberg, Germany). The results are expressed as the mean viability percentage of the treated compared to untreated control. The median effective concentration (EC_50_) was determined by sigmoidal regression curves using the GraphPad Prism 6.0 software. Additionally, the remaining live promastigotes were resuspended in Grace′s insect media and counted daily in a Neubauer chamber for three days.

### 4.5. Transmission Electron Microscopy (TEM)

After treatment with DODAB, parasites were centrifuged at 230× *g* for 10 min and the pellet was washed twice with 0.1 M of ammonium acetate, pH 7.0 (Sigma). Morphological changes in the parasites were analyzed by TEM after the preparation of thin epoxy resin-embedded sections containing parasites. For this, the treated promastigotes were fixed in 2.5% glutaraldehyde, 4% paraformaldehyde in a 0.1 M sodium cacodylate buffer (pH 7.4), washed in the same buffer and post-fixed in 1% osmium tetroxide. After dehydration in acetone series, the samples were embedded in Epon resin. Ultrathin sections were obtained with a Sorvall Ultramicrotome, stained with uranyl acetate and lead citrate and visualized in a transmission electron microscope operating at 80 kV. Images were recorded with an electron microscope Jeol EM 1011 (Jeol, Tokyo, Japan) and Gatan 785 ES1000W Erlangshen camera.

### 4.6. BMM Isolation

Bone marrow-derived macrophages (BMM) were isolated from femurs of 8–10 weeks old female C57BL/6 mice. Briefly, the mice were euthanized in a CO_2_ chamber. The femurs and tibias were then withdrawn and immersed in 70° GL alcohol for 2 min, followed by immersion in PBS. Afterwards the two epiphyses were removed and the bone flushed with 5mL of RPMI 1640 medium. The cell-containing flow-through was collected. The cell suspension was centrifuged at 230× *g* for 10 min at 4 °C and next resuspended in RPMI 1640 medium supplemented with 20% of FBS, HEPES (10 mM), sodium bicarbonate (1.5 g/L), sodium pyruvate (1 mM), penicillin G (100 U/mL), streptomycin (100 µg/mL), and 20% of L929 cell supernatant. Cells were cultured in cell culture dishes of 100 mm in diameter at 4 × 10^6^ cells in 10 mL of medium and incubated at 37 °C for 4 days in the presence of 5% CO_2_. On the fourth day, a further 10 mL of RPMI 1640 medium supplemented with 20% FBS and 20% supernatant of L929 cells was added to the plate. On the seventh day of culture, the adhered macrophages were detached by scraping with a cell scraper, and the cell viability was assessed using trypan blue (0.04%) and a hemocytometer (Neubauer chamber).

### 4.7. In Vitro Infection of BMMs with DODAB Pre-Treated Parasites

A total of 2 × 10^5^ BMM were seeded on glass coverslips in 24-well plates 24 h before experiments. The cells were incubated with DODAB pre-treated amastigotes (MOI 2) and promastigotes (MOI 5) in RPMI media supplemented with 10% FBS and 5% L929 cell supernatant for 2 h. Subsequently, the cells were washed three times with PBS to remove non-internalized parasites and incubated for 48 h at 34 °C. Infected cells were then fixed with ice-cold methanol at 100%. For immunofluorescence assays, methanol-fixed cells were blocked with BSA/TBS (0.1%) and incubated with polyclonal rat anti-mouse LAMP-1 (BD Bioscience, Franklin Lakes, USA) for 2 h, followed by incubation with goat anti-rat IgG antibody conjugated to Alexa 568 (Molecular Probes, Eugene, USA) to visualize the parasitophorous vacuole. To detect parasites, the samples were incubated with rabbit polyclonal anti-*Leishmania* antibodies followed with anti-mouse IgG conjugated to Alexa 488 (Molecular Probes) and 10 µg/mL of 4′,6-diamidino-2-phenylindole (DAPI; Sigma-Aldrich) for nuclei staining. Images were acquired by fluorescence microscopy (DMI6000B/AF6000-DFC365FX). The number of intracellular parasites was quantified by the blind direct counting of total number of macrophages, infected macrophages, and the total number of intracellular parasites. Two observers performed the counting with the assistance of the fluorescence microscopy. The results are expressed as the number of parasites per number of infected BMMs. At least 300 cells per coverslip were counted. The parasite load was determined by counting the number of intracellular parasites in at least 100 infected cells. The infection index was obtained by multiplying the percentage of infection per the average number of intracellular parasites per cell. The quantification of the PV size was performed by measuring the diameter in µm using the Image J software. At least 33 independent PV measurements were obtained.

### 4.8. Statistical Analysis

Data were analyzed with commercial software (GraphPad Software 6.0). Differences between means were analyzed by Student’s *t*-test. *p*-values of 0.05 or less were considered significant.

## Figures and Tables

**Table 1:** Differential in vitro cytotoxicity of DODAB.

Cell Type	Cells/mL	EC50 [DODAB]	Reference
**Mammalian cells**			
Kidney epithelial cells	105	5.4 mM	[19]
3T3(cloneA31) fibroblasts	104	1.0 mM	[18]
SV40- SVT2 fibroblasts	104	1.0 mM	[18]
**Gram-negative bacteria**			
E. coli	2 × 107	28 µM	[16]
S. typhimurium	2 × 107	10 µM	[16]
P. aeruginosa	3 × 107	5 µM	[16]
**Gram-positive bacteria**			
S. aureus	3 × 107	6 µM	[16]
**Yeasts**			
C. albicans ATCC 90028	2 × 107	10 µM	[17]
**Protozoa**			
Leishmania amazonensis *	1 × 106	25 µM	This work.

* 2 h of treatment, the other organisms were treated for 1 h.
